# Supplementary material for: Development of an In Vitro Biopotency Assay for an AAV8 Hemophilia B Gene Therapy Vector Suitable for Clinical Product Release
Source: Mol Ther Methods Clin Dev. 2020 Mar 17;17:581–8. doi: 10.1016/j.omtm.2020.03.013 (PMC7139127; doi:10.1016/j.omtm.2020.03.013)
Supplement: Document S1. Figures S1–S3 and Table S1–S3 [file mmc1.pdf]

OMTM, Volume *17*

Supplemental Information

Development of an *In Vitro* Biopotency Assay for an AAV8 Hemophilia B Gene Therapy Vector Suitable for Clinical Product Release

Johannes Lengler, Sogue Coulibaly, Bernadette Gruber, Reinhard Ilk, Josef Mayrhofer, Friedrich Scheiflinger, Werner Hoellriegl, Falko G. Falkner, and Hanspeter Rottensteiner

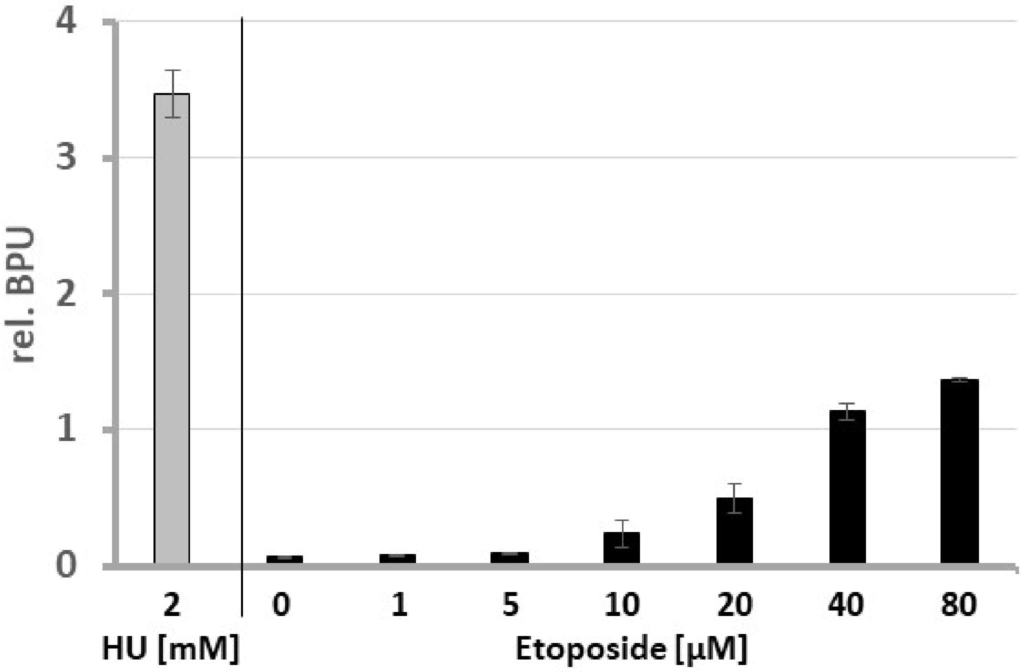

Supplementary Figure 1: Effect of etoposide on the *in vitro* biopotency assay for BAX 335. HepG2 cells were infected with the AAV8-FIX vector (MOI: $3x10^3$) in the presence of increasing concentrations of etoposide or 2 mM HU. After 4 days, FIX activity was measured in the supernatant. The observed increase in FIX activity with higher etoposide concentrations showed saturation at 80 µM and was less pronounced than that obtained with 2 mM HU.

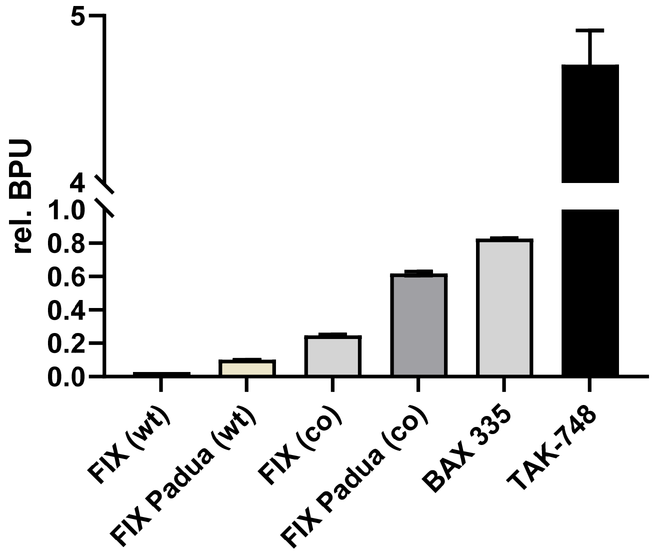

Supplementary Figure 2. Testing of other AAV8-FIX vectors with the *in vitro* biopotency assay for BAX 335. HepG2 cells were infected with the indicated AAV8-FIX vectors at a MOI dosage of $3x10^3$. After 4 days, FIX activity was measured in the supernatant. The expression cassettes of the 'wt' pair of vectors resemble that of the self-complementary vector BAX 335, but contain the normal human *F9* coding sequence, with or without the FIX Padua mutation. The expression cassettes of the 'co' vector pair are identical to those of the 'wt' pair, but contain a codon optimized and CpG-depleted *F9* coding sequence. The second generation AAV8-FIX vector TAK-748 has a single-stranded genome, a codon optimized and CpG-depleted *F9*-Padua coding sequence, and a novel liver-specific promoter. co, codon optimized; MOI, multiplicity of infection; Rel. BPU, relative biopotency unit; wt, wild-type.

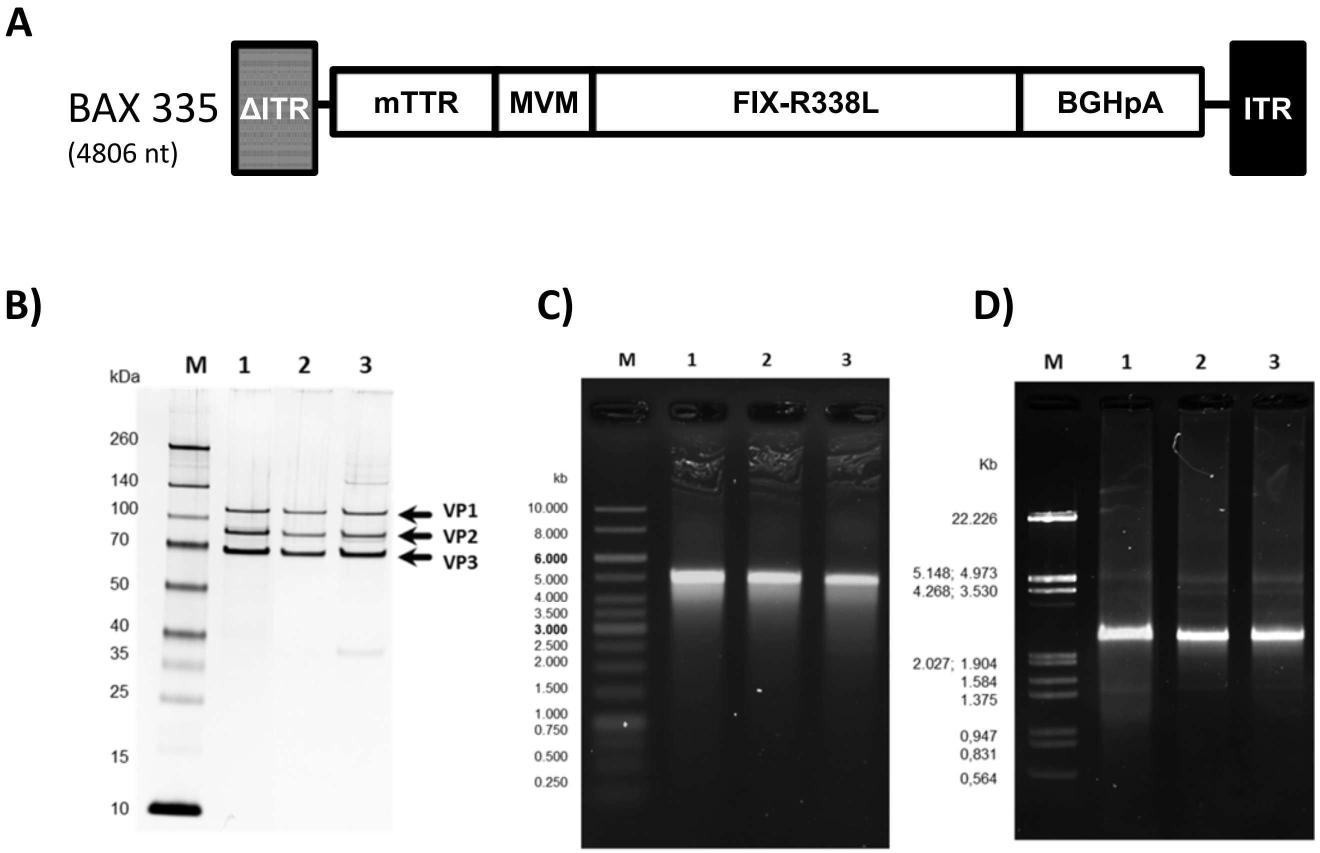

Supplementary Figure 3: Structural analysis of three lots of BAX 335. (A) Schematic drawing of the BAX 335 (scAAV8-FIX-R338L) self-complementary vector genome. **(B)** SDS-PAGE followed by silver staining. Analysis of three BAX 335 lots revealed the expected pattern of capsid proteins VP1, VP2 and VP3. **(C)** Alkaline agarose gel electrophoresis. Shown is the melted vector genome of the same three lots, with a single-stranded size of about 4.8 kb. **(D)** Native agarose gel electrophoresis. Shown are the vector genomes of the same three lots, running as a double stranded entity at a size of about 2.4 kb. The vector preparations show high purity and vector genome integrity. Lane M denotes respective size markers.

Supplementary Table 1: Influence of sample matrix components on *in vitro* biopotency assay specificity

Formulation buffer [%]	Mean BPU (n=2) Run 1	% Recovery	Mean BPU (n=2) Run 2	% Recovery	Mean BPU (n=2) Run 3	% Recovery	Mean BPU (n=6) All runs	% Recovery
1.25	0.84	99.1	0.89	85.6	0.82	92.7	0.85	92
1.00	0.84	99.1	0.89	86.2	0.78	88.2	0.84	90.8
0.75	0.86	101.1	0.96	93.1	0.88	99.9	0.9	97.7
0.50	0.93	109.6	0.97	93.8	0.91	102.7	0.94	101.5
0.25	0.99	116.3	0.92	88.5	0.83	93.9	0.91	98.8
0.00	0.85	100.0	1.03	100	0.88	100.0	0.92	100.0

Supplementary Table 2: Linearity of *in vitro* biopotency assay reference curve

Run	R^2
1	0.9933
2	0.9941
3	0.9964
4	0.9980
5	0.9960
6	0.9978
7	0.9900
8	0.9992
9	0.9945
10	0.9939
11	0.9981
12	0.9902
13	0.9979
14	0.9982
15	1.000
mean	0.9958
SD	0.0031

Supplementary Table 3a: Influence of different WCBs and cell passages on *in vitro* biopotency*

		BPU of control 1		Acceptance criteria [BPU]	
Day of cultivation	Cell passage number	WCB 1 (CS308)	WCB 2 (GR202)	Lower limit: 0.3063	Upper limit: 0.8614
11	13	0.51	0.56	pass	
18	15	0.40	0.59	pass	
25	17	n.a.	0.61	pass	
46	23	0.74	n.a.	pass	
53	25	0.56	0.55	pass	
60	27	0.61	n.a.	pass	

*Infection with small-scale AAV8-FIX vectors

Supplementary Table 3b: Influence of different WCBs and cell passages on *in vitro* biopotency*

		BPU of control 2		Acceptance criteria [BPU]	
Day of cultivation	Cell passage number	WCB 1 (CS308)	WCB 2 (GR202)	Lower limit: 0.3434	Upper limit: 0.7316
11	13	0.50	0.48	pass	
18	15	0.43	0.46	pass	
25	17	n.a.	0.49	pass	
46	23	0.59	n.a.	pass	
53	25	0.46	0.49	pass	
60	27	0.57	n.a.	pass	

*Infection with crude AAV8-FIX conditioned medium

Supplementary Table 3c: Influence of hydroxyurea on *in vitro* biopotency assay

BPU of control 1		Acceptance criterion [BPU]	
HU Lot 1	HU Lot 2	Lower limit: 0.3063	Upper limit: 0.8614
0.60	0.78	pass	
0.61	0.61	pass	
0.62	0.60	pass	
0.65	0.62	pass	

Supplementary Table 3d: Influence of hydroxyurea on *in vitro* biopotency assay

BPU of control 2		Acceptance criterion [BPU]	
HU Lot 1	HU Lot 2	Lower limit: 0.3434	Upper limit: 0.7316
0.47	0.56	pass	
0.46	0.49	pass	
0.47	0.52	pass	
0.50	0.51	pass	
