# Supplementary material for: Development of an In Vitro Biopotency Assay for an AAV8 Hemophilia B Gene Therapy Vector Suitable for Clinical Product Release
Source: Mol Ther Methods Clin Dev. 2020 Mar 17;17:581–8. doi: 10.1016/j.omtm.2020.03.013 (PMC7139127; doi:10.1016/j.omtm.2020.03.013)
Supplement: Document S2. Article plus Supplemental Information [file mmc2.pdf]

Molecular Therapy
Methods & Clinical Development
Original Article

Development of an *In Vitro* Biopotency Assay for an AAV8 Hemophilia B Gene Therapy Vector Suitable for Clinical Product Release

Johannes Lengler,[1] Sogue Coulibaly,[1] Bernadette Gruber,[1] Reinhard Ilk,[2] Josef Mayrhofer,[1] Friedrich Scheiflinger,[1] Werner Hoellriegl,[1] Falko G. Falkner,[1] and Hanspeter Rottensteiner[1]

[1]Baxalta Innovations GmbH, a member of the Takeda group of companies, Uferstraße. 15, A-2304 Orth an der Donau, Austria; [2]Baxter AG, a member of the Takeda group of companies, Industriestraße 67, A-1221 Vienna, Austria

Gene therapy product release requires reliable and consistent demonstration of biopotency. In hemophilia B vectors, this is usually determined *in vivo* by measuring the plasma levels of the expressed human factor IX (FIX) transgene product in FIX knockout mice. To circumvent this laborious assay, we developed an *in vitro* method in which the HepG2 human liver cell line was infected with the vector, and the resulting FIX activity was determined in the conditioned medium using a chromogenic assay. The initial low sensitivity of the assay, particularly toward adeno-associated viral serotype 8 (AAV8), increased approximately 100-fold and allowed linear measurement in a broad range of multiplicities of infection. Statistical parameters indicated high assay repeatability (relative standard deviation (RSD) < 5%) and intra-assay reproducibility (RSD < 20%). To compare the performance of the *in vitro* and *in vivo* biopotency assay, we applied statistical analyses including regression techniques and variation decomposition to the results obtained for 25 AAV8-FIX vector lots (BAX 335). These showed a highly significant correlation, with the cell culture-based assay demonstrating less variation than the *in vivo* test. The *in vitro* assay thus constitutes a viable alternative to using animals for lot release testing.

INTRODUCTION

Hemophilia B is caused by an X-linked mutation in the factor IX (FIX) (*F9*) gene, which encodes the circulating plasma coagulation FIX and occurs in approximately 1/25,000 male births.[1] Individuals lacking adequate amounts of FIX in the circulation are at increased risk for spontaneous bleeding. Current therapy for hemophilia B involves regular infusions of FIX protein concentrates to prevent and treat bleeding events. These concentrates must be administered intravenously on a regular basis throughout the patient's lifetime.[2] In recent years, gene therapy using adeno-associated viral (AAV) vectors has shown promise in treating hemophilia B because of the viral vectors' relative safety and long-term gene expression.[3] Applying AAV serotype 8 (AAV8) vectors carrying a codon-optimized and CpG-depleted human *F9* gene in clinical studies has led to a substantial increase in FIX levels, reducing or even obviating the need for recombinant FIX administration.[4] The efficacy of hemophilia B gene therapy could be improved upon employing the single amino acid exchange variant FIX Padua, where leucine is substituted for arginine at position 338 (R338L).[5] This naturally occurring variant shows an up to 10-fold higher specific activity than wild-type (WT) FIX[6] and yields adequate FIX activity despite low protein expression levels.[7]

Demonstration of biopotency for clinical-grade AAV-FIX vectors is a release criterion and is typically achieved by *in vivo* analysis in mice.[8] Nonetheless, for AAV product release, the US Food and Drug Administration (FDA) recommends establishing an adequate *in vitro* biopotency assay to be refined and qualified during clinical development and validated for Biologics License Application (BLA) submission.[9] Implementation of an *in vitro* rather than an *in vivo* biopotency assay would also lower costs and reduce efforts to run the assay, as well as minimize the number of animals used in line with the principles of the 3 Rs.[10] Previous attempts to set up a FIX *in vitro* biopotency assay based on human liver cell line infection were thwarted by low sensitivity, particularly for AAV8, and thus *in vitro* analysis of FIX gene therapy vectors was circumvented where possible.[8,11,12]

Here we describe the development of a highly sensitive *in vitro* biopotency assay for BAX 335, an AAV8-based hemophilia B gene therapy vector expressing FIX Padua that was explored in a clinical phase I/II study (ClinicalTrials.org: NCT01687608). The assay underwent several optimization steps and was evaluated for its linearity, reproducibility, and specificity. Finally, a thorough statistical analysis was used to compare its performance with that of the standard *in vivo* biopotency assay.

RESULTS

Development of an *In Vitro* Biopotency Assay for BAX 335

BAX 335 is an AAV8-based hemophilia B gene therapy vector with a self-complementary vector genome and an expression cassette

Received 19 February 2020; accepted 12 March 2020;
https://doi.org/10.1016/j.omtm.2020.03.013.

Correspondence: Hanspeter Rottensteiner, Baxalta Innovations GmbH, a member of the Takeda group of companies, Uferstraße. 15, A-2304 Orth an der Donau, Austria.
E-mail: Hanspeter_.Rottensteiner@chello.at

© 2020 Shire International GmbH, now part of Takeda, Zurich, Switzerland.
This is an open access article under the CC BY license (http://creativecommons.org/licenses/by/4.0/).

Table 1. FIX Activity Depends on MOI, Cell Number, and Incubation Time

Experiment	Cells Seeded ($\times 10^5$/mL)	Time (Days)	BAX 335 MOI	FIX Activity (%)
A	1.3	1	5.0E+5	0.003
	1.3	2	5.0E+5	0.04
	1.3	3	5.0E+5	0.18
	1.3	6	5.0E+5	1.8
B	1.3	6	1.3E+5	0.37
	1.3	6	2.5E+5	0.57
	1.3	6	5.0E+5	0.91
C	2.5	4	1.3E+5	0.26
	2.5	4	2.5E+5	0.64
	2.5	4	5.0E+5	1.2

designed to express a codon-optimized FIX Padua transgene from the liver-specific transthyretin (TTR) promoter/enhancer combination.[13,14] A liver-derived cell line that is amenable for infection by AAV8 was needed to analyze the biological function of BAX 335 *in vitro*. We thus selected the human HepG2 cell line, which was successfully used for AAV2 infections in past studies.[15,16]

The initial sensitivity of the assay was low, requiring multiplicities of infection (MOIs) of 5×10^5 and higher to detect any FIX activity (data not shown). This is in line with previous reports describing low *in vitro* biopotencies for liver-targeted gene therapy vectors.[11–13] We therefore sought to optimize the assay performance examining several parameters. To save vector material, we first determined the smallest possible format for the infections. This turned out to be a 48-well plate, which yielded just enough cell culture supernatant volume (250 μL) for reliable FIX activity testing. We then tested the effect of incubation time, vector dose, and number of cells per well on assay performance (Table 1). Experiments A–C showed that (experiment A) measurable FIX activity in the supernatant increased over time; (experiment B) FIX expression was dose dependent; and (experiment C) by increasing the cell density from 1.25×10^5 to 2.5×10^5 cells/mL, 4 days of incubation was sufficient to achieve substantial FIX expression levels (experiment C).

To further decrease the infection dose per cell (i.e., the MOI), four substances described to enhance infection were investigated in our assay system: the proteasome inhibitor MG132, the chemotherapeutic drug arsenic trioxide,[17] the DNA synthesis inhibitor hydroxyurea (HU),[18] and the topoisomerase II inhibitor etoposide.[19] Whereas MG132 and arsenic trioxide failed to increase BAX 335-mediated FIX expression in HepG2 cells (data not shown), pretreatment of cells with HU boosted infection over a concentration range of 1–10 mM (Figure 1A). FIX expression peaked at a concentration of 2 mM HU, where FIX activity was 20-fold higher than in the preparation without HU. An increase in activity was observed at three MOIs (2.75×10^3, 5.50×10^3, and 1.10×10^4), indicative of a robust effect of this compound. The topoisomerase II inhibitor etoposide, reported

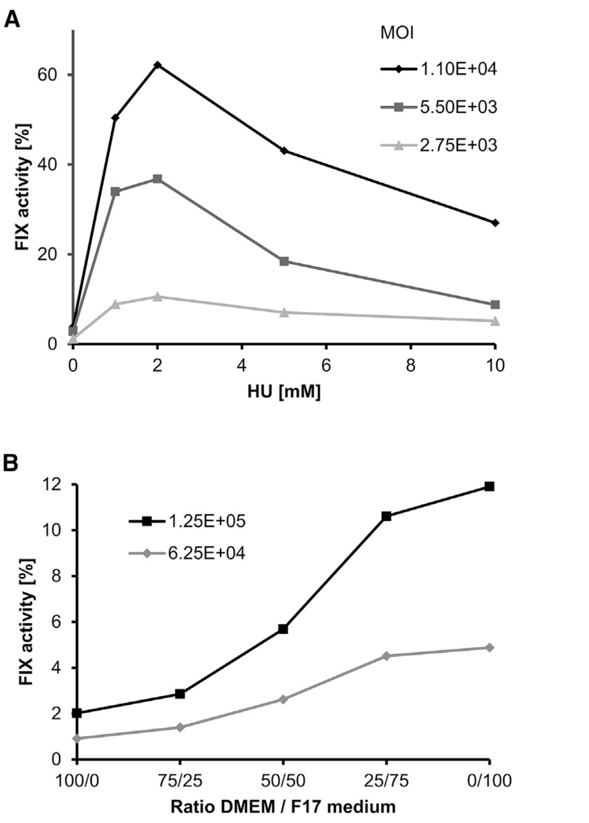

Figure 1. Optimization of the *In Vitro* Biopotency Assay for BAX 335

(A) Effect of HU on BAX 335-mediated FIX expression. HepG2 cells were infected with the AAV8-FIX vector at the indicated MOI dosages in the presence of increasing concentrations of HU. After 4 days, FIX activity was measured in the supernatant. FIX activity peaked at a concentration of 2 mM HU. (B) Influence of cell culture medium composition on FIX expression. HepG2 cells were infected with BAX 335 at two MOI dosages in mixtures of DMEM/FCS and synthetic FreeStyle F17 culture media. After 4 days, FIX activity was measured in the supernatant. Increasing the proportion of FreeStyle F17 medium continuously improved FIX expression. DMEM, Dulbecco's modified Eagle's medium; FCS, fetal calf serum; HU, hydroxyurea; MOI, multiplicity of infection.

to increase AAV transduction frequency of human fibroblasts to a similar extent as HU,[19] also enhanced FIX expression in our setting (concentration range: 1–80 μM), but the improvement was not as pronounced as with HU (Figure S1). At the highest concentration tested, etoposide began to affect cell growth and viability, preventing a further concentration increase.

Finally, a suitable cell culture medium for cell infection and transgene expression was identified in a titration experiment with the previously used Dulbecco's modified Eagle's medium with fetal calf serum (DMEM/FCS) medium and FreeStyle F17 (both Thermo Fisher Scientific). The latter medium is a proprietary expression medium for mammalian cells that does not contain animal-derived components but whose composition is not disclosed by the supplier (Thermo Fisher Scientific). The two media were mixed at different ratios and

Table 2. Intra-assay Recovery of *In Vitro* Biopotency Assay

	Run 1		Run 2		Run 3	
MOI	Mean BPU (n = 2)	% Recovery	Mean BPU (n = 2)	% Recovery	Mean BPU (n = 4)	% Recovery
3.27E+3[a]	0.59	100.0	0.88	100.0	0.65	100.0
1.64E+3	0.57	97.6	0.73	83.3	0.78	119.5
6.54E+3	0.58	98.4	0.84	95.7	0.64	97.7

[a]Reference dose.

tested for their effect on HepG2 cell infection by BAX 335. Expression of FIX increased with increasing FreeStyle F17 medium content and was over 5-fold higher with pure FreeStyle F17 medium (Figure 1B).

The combined assay optimization data allowed us to define standard conditions for the *in vitro* biopotency assay, where HepG2 cells (2.5×10^5 cells/mL or 6.25×10^4 cells/well) are pretreated with 2 mM HU prior to infection with BAX 335 in FreeStyle F17 medium, followed by an incubation period of 4 days. With this setup, the assay was sufficiently sensitive for efficient and reliable testing of BAX 335 vector preparations at reasonably low doses (MOI < 10^4).

We further assessed whether the assay was useful for testing other AAV8-FIX vectors. For this purpose, we compared BAX 335 with two pairs of otherwise identical self-complementary vectors designed to express WT FIX or FIX Padua, using either the WT or a codon-optimized and CpG-depleted *F9* nucleotide sequence (Figure S2). As expected, the WT *F9* nucleotide sequence yielded the lowest FIX expression levels. Introduction of the R338L FIX Padua mutation increased expression 3-fold. Expression was approximately 7.5-fold increased for the codon-optimized pair of vectors, again with a 3-fold higher biopotency for the Padua variant. Compared with BAX 335, FIX expression from the CpG-reduced, codon-optimized sequence (co-R338L) was in the same range, whereas that from the WT nucleotide sequence (WT-R338L) was lower.

Next, we compared expression of BAX 335 with a second generation vector (TAK-748) developed after the interim readout of the BAX 335 clinical study, which revealed an immunological issue that resulted in a loss of FIX expression in all but one participant after 5–11 weeks.[20] This vector is single-stranded with a codon-optimized and CpG-depleted *F9* Padua cDNA and a stronger liver-specific promoter. The assay showed a 5.7-fold increase in FIX expression for the new vector (Figure S2), commensurate with improved potency.

Taken together, the FIX *in vitro* biopotency assay reflected the expected potency ranking for a number of constructs, suggesting its broad applicability for AAV8-FIX vectors.

Reliability of the *In Vitro* Biopotency Assay

To ensure reliable measurement of the vectors' *in vitro* biopotency, we subjected our assay system to a qualification process, addressing accuracy, precision, specificity, linearity, range, and robustness. Because an international standard is lacking, accuracy was assessed as FIX activity recovery using a purified BAX 335 preparation at different MOIs. Intra-assay recovery, calculated from three assay runs, was 83%–120% (Table 2), and inter-assay recovery, determined from the mean of the same three assay runs, was 97%–99% (Table 3). Replicate testing of a single lot under standard conditions by one analyst on the same day resulted in a relative standard deviation (RSD) below 5%, demonstrating precision of the assay (Table 4).

Testing seven further lots and performing the assay on three different days using different equipment resulted in an RSD of <19% (Table 5, lot numbers 2–7). Inclusion of a second analyst increased the RSD to approximately 23% (Table 5, lot number 8). The combined results indicated that the assay performed with an intermediate precision that is acceptable for complex biological assays.[21]

Specificity was addressed by assaying BAX 335 samples with increasing volumes of formulation buffer to test for matrix effects. Because tested samples usually comprise less than 0.2% of the assay volume, samples were spiked with an additional volume of up to 1.25% of formulation buffer. Recovery of *in vitro* biopotency activity remained constant compared with that of non-spiked samples (Table S1). Thus, the assay had an acceptable specificity for samples with a titer of 2.6E+11 vg/mL or higher.

Linearity of the assay was investigated by infecting HepG2 cells under standard conditions with six dilutions of BAX 335 vector material, resulting in MOIs of 8.18E+2 to 6.55E+3. A highly correlated dose response with a coefficient of determination (R^2) above 0.99 was obtained, suggesting that the assay range covers at least these MOIs (Table S2).

Robustness of the assay system was tested for two critical parameters: HepG2 cell passage number and HU lot-to-lot variability. Two different working cell banks at increasing cell passage numbers

Table 3. Inter-assay Recovery of *In Vitro* Biopotency Assay

MOI	Mean BPU (n = 3)	SD	% RSD	% Recovery
3.27E+3[a]	0.71	0.15	21.73	100.0
1.64E+3	0.69	0.11	15.71	98.4
6.54E+3	0.68	0.14	20.19	97.0

[a]Reference dose.

Table 4. Repeatability of *In Vitro* Biopotency Assay

Sample Lot	Mean BPU (n = 12)	SD	RSD (%)
1	0.36	0.017	4.72

were used to determine the biopotency of two different control samples, a small-scale AAV8-FIX preparation (Table S3A) and a crude AAV8-FIX preparation containing conditioned medium of the fermentation process (Table S3B). The two working cell banks revealed comparable results for both controls. In addition, no potency decrease was observed from day 11 to day 60, corresponding to an increase in passage number from 13 to 27. Individual measurements for all samples were within the acceptance criteria as determined by Minitab. Activities of both control samples were further compared with respect to two lots of the cell infection enhancer HU, and individual measurements of BPU were again within the acceptance criteria (Tables S3C and S3D).

In summary, our assay conditions yielded highly robust FIX *in vitro* biopotency results.

Comparison of the *In Vitro* and *In Vivo* Biopotency Assay

For lot release of hemophilia B gene therapy products, a mouse *in vivo* biopotency assay is commonly used. We also employed this assay for preclinical and phase I/II clinical lot release to ensure that the vector product translates into FIX activity in the circulation. However, because this assay is laborious and medical authorities are recommending *in vitro* studies where appropriate,[22] we evaluated whether our newly developed *in vitro* biopotency assay could consistently deliver comparable results with the *in vivo* test and eventually replace it for lot release testing.

For this purpose, 25 BAX 335 vector lots encompassing small- and large-scale preparations of different purity were repeatedly tested in both assays, and average FIX activity values were calculated for each lot. The methods demonstrated highly similar behavior, i.e., a low (or high) biopotency in the *in vivo* assay corresponded to a low (high) biopotency in the *in vitro* assay (Figure 2A).

Table 5. Inter-assay Variability of *In Vitro* Biopotency Assay

Lot	Mean BPU	SD	RSD (%)	Replicates	Experiments	Total Data Points
2	1.11	0.172	15.52	2	3	6
3	0.80	0.135	16.81	2	3	6
4	0.45	0.079	17.50	2	3	6
5	0.51	0.084	16.34	2	3	6
6	0.44	0.068	15.49	2	3	6
7	0.65	0.121	18.61	2	3	6
8	0.78	0.044	5.68	2	27	54
9[a]	0.91	0.206	22.60	2–5	4	24

[a]Assays performed by two different analysts.

Analysis of variance demonstrated discrimination of samples, with no indication of a mismatch between the two methods (p for method/sample interaction = 0.135). Correlation analysis confirmed a highly significant relationship between the lots' average activity values for both methods, as shown by a Pearson correlation coefficient of 0.926 with a p value <0.001 (Figure 2B).

We then compared over 350 individual data points for the distribution of the single measurements with the average of all values generated for each lot by test method. Analysis of these residuals showed less variation for the *in vitro* than the *in vivo* test, reflected by a lower deviation of the single measurements from their respective average value (Figure 2C). Outliers to the bottom were observed for some *in vivo* data points and probably reflect non-responders in mice.

Taken together, our method comparison showed accordance of the two assays, and thus justifies replacing the *in vivo* biopotency test with the *in vitro* assay.

DISCUSSION

A key regulatory requirement for the clinical use of gene therapy vectors is demonstration of their biopotency. This can either be accomplished by *in vivo* testing, preferably using an indication-specific animal model, or by using a cell-type-specific *in vitro* assay.[22,23] Although *in vitro* biopotency assays are recommended by regulatory authorities, their use in clinical lot release has been limited by inefficient transduction of appropriate immortalized cell lines and the questionable relevance of such assays in the absence of a clear correlation with the results obtained using an established animal model.

Here we describe the development of an optimized HepG2 cell-based *in vitro* assay for quantification of FIX expression as a direct measure of the biopotency of BAX 335, an AAV8-based gene therapy vector designed to treat hemophilia B patients. The final setup allowed a reduction in the higher than 5×10^5 MOI infection dose by at least two logs compared with the starting setup, resulting in a MOI of 3×10^3. Published data on *in vitro* biopotency assays using HepG2 cells indicate that for AAV2-based vectors, FIX expression measurement requires MOIs of 2×10^5 for single-stranded and 2×10^4 for self-complementary vectors.[11,13] The obtained sensitivity for AAV vectors of serotype 8 used here is even lower and has been described to be particularly inefficient in the context of an AAV8-FIX vector.[8] Osman et al.[12] reported a MOI of $1.6–3.2 \times 10^5$ for the *in vitro* infection of HepG2 cells using a self-complementary AAV8 vector expressing the apoE3 transgene. The sensitivity of single-stranded AAV8 vectors is expected to be a further log lower.[14]

Optimization procedures for the FIX biopotency assay focused on choice of cell culture medium and compounds that might boost vector transduction. Switching from DMEM to the richer FreeStyle F17 medium resulted in a 5-fold increase in FIX expression. The latter medium's composition was shown to promote transgene expression in HepG2 cells and may have also stabilized the FIX transgene product in the supernatant.

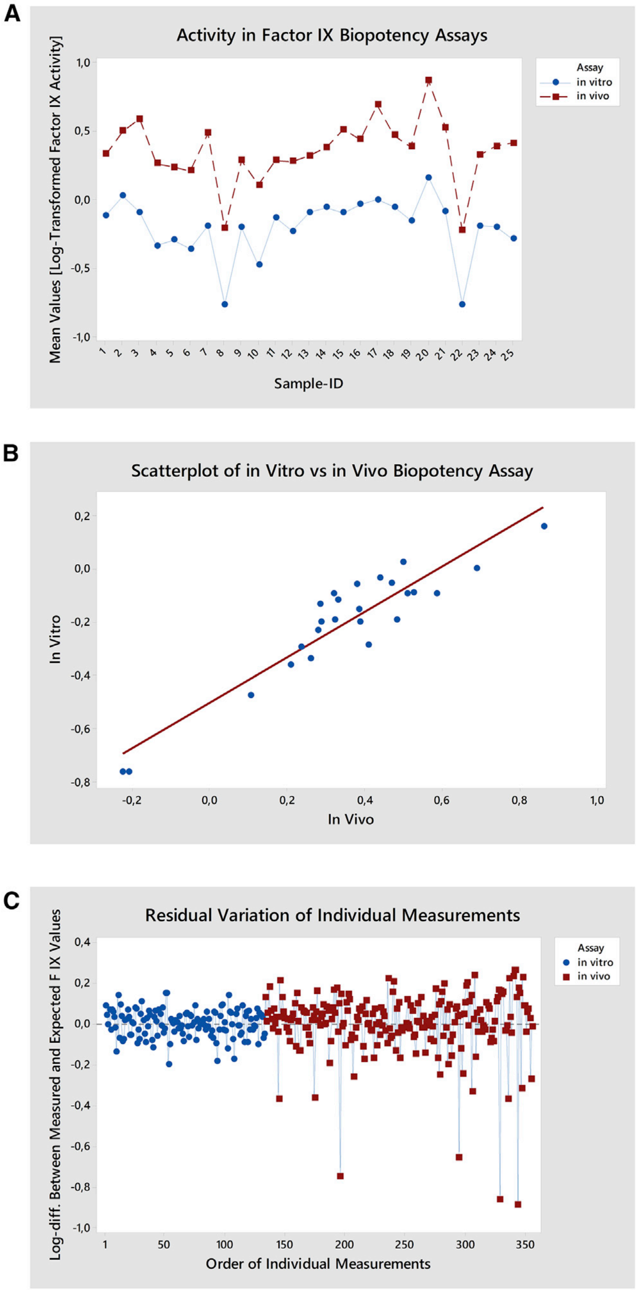

Figure 2. Correlation of the Results for the BAX 335 *In Vitro* and *In Vivo* Biopotency Assays

(A) Assay comparison with 25 BAX 335 lots. The lots were tested with the *in vitro* and *in vivo* biopotency assay for FIX expression, and resulting averages were compared in an interaction plot at a logarithmic scale. The methods showed highly similar behavior. (B) Statistical analysis of data correlation. The average *in vitro* and *in vivo* biopotencies of the 25 BAX 335 lots were plotted as log FIX activities against each other. A linear dependency was observed, with a Pearson correlation of 0.926 and a p value <0.001. (C) Statistical analysis of residuals. Single measurement values were analyzed for their distribution from their respective average of each lot. Deviations are plotted as x-fold of the standard deviation. Deviation of data points from the *in vivo* biopotency assay was more pronounced with some downward outliers discernible, indicating that the *in vitro* assay performed more robustly than the *in vivo* assay.

Another significant improvement was achieved by supplementing the medium with 2 mM HU, which led to an approximately 20-fold increase in FIX expression. HU was previously reported to enhance transduction and mobilize virions into the nucleoplasma.[18] This compound prevents DNA synthesis by inhibiting ribonucleotide reductase lowering the cellular pools of deoxynucleotides.[24] At the same time, unscheduled DNA repair mechanisms are induced; therefore, HU's stimulating effect on AAV transduction was largely ascribed to increasing conversion of single-stranded vector genomes to transcriptionally active double-stranded molecules.[19,25] We observed a 20-fold higher transduction for a self-complementary AAV8 vector. One possible explanation is the observed retarded cell division over the 4 days of incubation, which prevented cellular outgrowth and may have minimized dilution of the non-integrated AAV vector genome, leading to accumulation of FIX levels in the supernatant. It is also likely that HU-triggered DNA repair mechanisms also promoted late-stage transduction events for self-complementary vector genomes. Similar considerations were put forward regarding the mode of action of the topoisomerase inhibitor etoposide.[26] In both cases, however, the underlying mechanism remains ill-defined.

Proteasome inhibitors have also been shown to improve the late stages of AAV transduction, leading to enhanced reporter gene expression.[27,28] In our hands, however, the proteasome inhibitor MG132 did not enhance FIX expression, possibly because AAV8 is less susceptible to the reported MG132-triggered transduction increase in our setup. The chemotherapeutic drug arsenic trioxide was described to cause an accumulation of AAV2 capsids at the perinuclear region in several cell lines, facilitating productive nuclear trafficking and improving transduction.[17] Testing this compound in our HepG2-based assay system, though, did not show an increase in FIX expression after infection with the AAV8-FIX vector BAX 335.

The reliability of the *in vitro* biopotency assay was assessed by analysis of validation relevant parameters. Its reproducibility fulfilled the US Pharmacopeia Convention for gene therapy products allowing variation of 30%–50%.[21] Our stringent assay design yielded variations of 23% or lower, thus establishing an effective tool for monitoring product quality. Under these conditions, high assay reproducibility was observed. Other qualification parameters confirmed the *in vitro* biopotency assay to be a robust assay system with a wide linear range.

Comparison of the measured *in vitro* biopotency values for 25 BAX 335 vector preparations with those of the widely used mouse *in vivo* biopotency assay demonstrated a strong correlation, as shown by a highly significant Pearson coefficient. Notably, the *in vitro* method was characterized by less variation in individual measurements. The variation observed using the *in vivo* assay was mainly caused by the low FIX activity measured for some plasma samples, due to non-responders and to responders that had formed anti-FIX antibodies

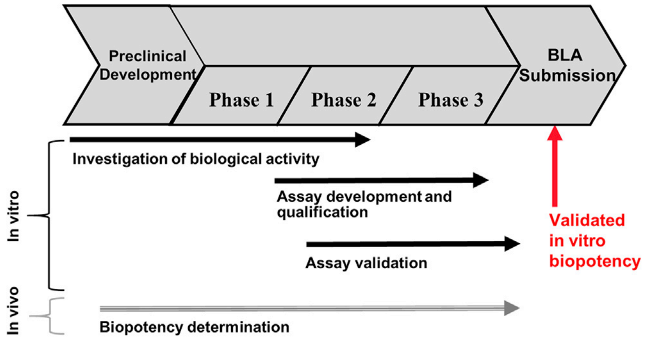

Figure 3. Timeline for *In Vitro* Biopotency Testing during Clinical Phases
An *in vivo* biopotency assay is initially used for product release. In parallel, *in vitro* biopotency is evaluated and an assay developed, to replace the *in vivo* assay after phase 3. Illustration was modified from a presentation by Gavin.[23]

causing a loss in plasma activity levels. These non-responders were rare (<5%) and distributed randomly over several vector preparations and studies.

Given the differing nature of the two biopotency detection systems, the observed matching behavior was unexpected. Although the mouse model mimics physiological conditions, its limitation lies in mice expressing a human xenoprotein in their hepatocytes. The *in vitro* model uses cells of human origin, but in a non-physiological, two-dimensional culture system. The two approaches thus have different implications on infectivity, transcription, and expression rates, and on the half-life of the transgene product. Furthermore, a chromogenic assay was used for the *in vitro* biopotency assay FIX activity, and a one-stage clotting assay for the *in vivo* biopotency assay.

The correlation between the two methods provides the rationale for switching from the *in vivo* to the *in vitro* biopotency assay for product release not only for BAX 335, but also for any other liver-targeted AAV8 vector. Because the FDA recommends rigorous comparison of methods in such cases,[23] we suggest running both assays in parallel until the requested dataset is obtained, and using only the *in vitro* biopotency assay upon market authorization (Figure 3).

MATERIALS AND METHODS

Characterization of BAX 335 Vector Material

Vector Design

Development of BAX 335 has been described previously.[29] The scAAV-FIX-R338L vector genome is composed of a promoter/enhancer combination derived from liver-specific mouse TTR (prealbumin, TTR) promoter,[30] a short intron fragment from minute virus of mice (MVM),[31] a codon-optimized FIX Padua (R338L) coding sequence,[5] and a polyadenylation signal derived from the bovine growth hormone (BGHpA). The expression cassette is flanked by AAV2 inverted terminal repeats (ITRs), with the 5′-ITR (mut 5′-ITR) being mutated to direct preferential replication and packaging of self-complementing rather than conventional single-stranded AAV DNA sequences (Figure S3A).

The AAV8-FIX vectors AAV8-FIX-WT and AAV8-FIX-WT-R338L were generated by replacing the codon-optimized *F9* coding sequence of BAX 335 with the WT *F9* nucleotide sequence, with (AAV8-FIX-WT-R338L) or without (AAV8-FIX-WT) the R338L mutation, or with a codon-optimized and CpG-depleted *F9* nucleotide sequence,[32] also with (AAV8-FIX-co2-R338L) or without (AAV8-FIX-co2) the R338L mutation. The second generation vector TAK-748 was further modified to contain a single-stranded genome, a newly codon-optimized and CpG-depleted *F9* nucleotide sequence with the R338L mutation, and a stronger liver-specific promoter.[33]

Manufacturing

Vectors were prepared by the triple-plasmid transfection method as described previously,[31] using HEK293 suspension cells and a cocktail of the BAX 335 expression plasmid, the helper plasmid pXX6-80 (carrying adenoviral helper genes), and the packaging plasmid pGSK2/8 (contributing the rep2 and cap8 genes). Of the 25 lots included in the analysis, 6 were generated at 1-L scale and processed according to Grieger et al.,[34] using an iodixanol density gradient followed by a one-step HiTrap Q HP anion exchange purification step. The other lots were produced at 200-L scale and generated by processing the conditioned medium via sequential application of Mustang Q anion exchange chromatography, density gradient ultracentrifugation, and TMAE anion exchange chromatography.[35] Research vectors AAV8-FIX-WT, AAV8-FIX-WT-R338L, AAV8-FIX-co2, AAV8-FIX-co2-R338L, and TAK-748 were prepared at 1-L scale according to Grieger et al.,[34] as described above. Vectors were quantified by qPCR using the ITR-qPCR procedure targeting the AAV2 ITRs common to all vectors.[36]

Purity and Genomic Integrity

All materials were controlled by protein and DNA gel analyses. The expected pattern for capsid proteins VP1, VP2, and VP3 was confirmed by SDS-PAGE applying standard procedures (Figure S3B). Each lane contained 1×10^{10} vg of the viral vector and was separated on a 4%–12% Bis-Tris gel (NuPAGE Novex, Life Technologies, Austria). Silver staining was performed with a SilverQuest kit (Novex, Life Technologies) according to the manufacturer's instructions.

The integrity of the vector genomes was analyzed by alkaline (Figure S3C) and native (Figure S3D) agarose gel electrophoresis. Alkaline gel electrophoresis, which melts the hybridized complementary strands to result in a single-stranded DNA-strand 2-fold in length, was run under alkaline conditions.[37] Approximately $1–2 \times 10^{10}$ vector genomes (vg) of AAV particles were loaded in the presence of SDS on an agarose gel. Following separation, the gel was incubated in Tris-HCl and finally stained with GelRed dye (Biotium, Austria).

Native electrophoresis was performed as described previously.[37] In brief, AAV vector preparations were incubated at 75°C for 10 min in the presence of 0.5% SDS and then cooled to room temperature. Approximately 1.5×10^{10} vector genomes (vg) were loaded per

lane on a 1% 1× TAE agarose gel and electrophoresed for 60 min at 7 V/cm of gel length. The gel was then stained in 2× GelRed (Biotium, Germany) solution and imaged by ChemiDocTMMP (Bio-Rad, Austria).

Description of the *In Vitro* Biopotency Assay

The assay was set up with the human liver cell line HepG2, obtained from ATCC (ATCC HB-8065). HepG2 cells were cultivated in DMEM with 10% FCS and supplemented with 2 mM HU (Sigma-Aldrich, Germany). One day later, cells were seeded in 48-well plates in F17 medium containing 350 ng/mL vitamin K_3 (Sigma-Aldrich), infected with BAX 335 at a MOI of 3×10^3, and incubated for 96 h. The amount of FIX secreted into the cell supernatant was quantified by determining the FIX chromogenic activity using the Rox Factor IX kit (Rossix, Moelndal, Sweden).

The standard curve was generated by transduction of HepG2 cells with a purified AAV8-FIX standard at MOIs of 7×10^2 to 7×10^3. The FIX activity measured with the standard at a MOI of 3×10^3 was defined as an arbitrary bio-potency unit (BPU). All measured FIX activities were normalized to this value and reported as relative BPUs. As controls, two purified BAX 335 vectors and a crude BAX 335 vector-containing supernatant were measured at the same MOI (3×10^3) in each assay run and monitored via control chart.

Description of the *In Vivo* Biopotency Assay

The *in vivo* biopotency assay was performed using FIX knockout (B6;129P2-$F9^{tm1Dws}$) mice[38] that were bred by Charles River (Sulzfeld, Germany) and kept as described previously.[39] Six to eight animals per group were administered 4×10^{11} vg/kg body weight of the respective vector preparation via tail vein injection. Blood was drawn 14 days after injection by retro-orbital puncture, and plasma was prepared and frozen using standard procedures. Human FIX activity in mouse citrate plasma was determined by the one-stage activated partial thromboplastin time (APTT) assay using human FIX-deficient plasma as a substrate essentially as described previously.[38]All animal experiments followed a protocol authorized by the Local Authorities on Animal Experiments or the Institutional Animal Care and Use Committee (IACUC).

Statistical Methods

All analyses were carried out using the statistical software package Minitab. In order to obtain homogeneous measurement variation despite the differences between samples and methods, data were log-transformed to scale relative differences uniformly across the analytical range. All hypothesis decisions were made based on a level of significance of $\alpha = 5\%$.

Hypotheses regarding the ability of the method to discriminate samples (main effect) and the uniformity of sample patterns (interaction) were tested using a general linear model (GLM). The degree of association between the two methods was analyzed using a correlation analysis testing Pearson correlation coefficient for significance.

SUPPLEMENTAL INFORMATION

Supplemental Information can be found online at https://doi.org/10.1016/j.omtm.2020.03.013.

AUTHOR CONTRIBUTIONS

J.L. designed the research, established the assay formats, performed the experiments, analyzed and interpreted the data, and wrote the manuscript. B.G. and J.M. performed the experiments. S.C. and W.H. designed and conducted the *in vivo* experiments. R.I. performed the statistical analysis. F.G.F. and F.S. interpreted the data and reviewed the manuscript for intellectual content. H.R. interpreted the data and wrote the manuscript.

ACKNOWLEDGMENTS

J.L., S.C., B.G., J.M., F.S., W.H., and H.R. are employees of Baxalta Innovations GmbH, a member of the Takeda group of companies; F.G.F. was an employee of Baxalta Innovations GmbH at the time of the study. R.I. is an employee of Baxter AG, a member of the Takeda group of companies. F.S., W.H., and H.R. are Takeda stock owners. We thank Karima Benamara for excellent editing of the manuscript. This research was funded by Baxalta Innovations GmbH, Austria, a member of the Takeda group of companies.

REFERENCES

1. Saini, S., and Dunn, A.L. (2019). Hemophilia B. In Transfusion Medicine and Hemostasis, Third Edition, B.H. Shaz, C.D. Hillyer, and M. Reyes Gil, eds. (Elsevier), pp. 685–690.
2. Stonebraker, J.S., Bolton-Maggs, P.H., Brooker, M., Farrugia, A., and Srivastava, A. (2011). A study of reported factor IX use around the world. Haemophilia *17*, 446–455.
3. High, K.A. (2001). AAV-mediated gene transfer for hemophilia. Ann. N Y Acad. Sci. *953*, 64–74.
4. Nathwani, A.C., Tuddenham, E.G., Rangarajan, S., Rosales, C., McIntosh, J., Linch, D.C., Chowdary, P., Riddell, A., Pie, A.J., Harrington, C., et al. (2011). Adenovirus-associated virus vector-mediated gene transfer in hemophilia B. N. Engl. J. Med. *365*, 2357–2365.
5. Simioni, P., Tormene, D., Tognin, G., Gavasso, S., Bulato, C., Iacobelli, N.P., Finn, J.D., Spiezia, L., Radu, C., and Arruda, V.R. (2009). X-linked thrombophilia with a mutant factor IX (factor IX Padua). N. Engl. J. Med. *361*, 1671–1675.
6. Weber, A., Engelmaier, A., Voelkel, D., Pachlinger, R., Scheiflinger, F., Monahan, P.E., and Rottensteiner, H. (2018). Development of Methods for the Selective Measurement of the Single Amino Acid Exchange Variant Coagulation Factor IX Padua. Mol. Ther. Methods Clin. Dev. *10*, 29–37.
7. Monahan, P.E. (2015). Gene therapy in an era of emerging treatment options for hemophilia B. J. Thromb. Haemost. *13* (*Suppl 1*), S151–S160.
8. Allay, J.A., Sleep, S., Long, S., Tillman, D.M., Clark, R., Carney, G., Fagone, P., McIntosh, J.H., Nienhuis, A.W., Davidoff, A.M., et al. (2011). Good manufacturing practice production of self-complementary serotype 8 adeno-associated viral vector for a hemophilia B clinical trial. Hum. Gene Ther. *22*, 595–604.
9. Office of Medical Products and Tobacco, Center for Biologics Evaluation and Research (2011). Potency tests for cellular and gene therapy products. U.S. Food and Drug Administration. January 2011, FDA-2008-D-0520, https://www.fda.gov/regulatory-information/search-fda-guidance-documents/potency-tests-cellular-and-gene-therapy-products.
10. Lang, C., Kolaj-Robin, O., Cirefice, G., Taconet, L., Pel, E., Jouette, S., Buda, M., Milne, C., and Charton, E. (2018). Replacement, Reduction, Refinement—Animal welfare progress in European Pharmacopoeia monographs: activities of the European Pharmacopoeia Commission from 2007 to 2017. Pharmeur. Bio Sci. Notes *2018*, 12–36.

11. Nathwani, A.C., Davidoff, A., Hanawa, H., Zhou, J.F., Vanin, E.F., and Nienhuis, A.W. (2001). Factors influencing in vivo transduction by recombinant adeno-associated viral vectors expressing the human factor IX cDNA. Blood *97*, 1258–1265.

12. Osman, E., Evans, V., Graham, I.R., Athanasopoulos, T., McIntosh, J., Nathwani, A.C., Simons, J.P., Dickson, G., and Owen, J.S. (2009). Preliminary evaluation of a self-complementary AAV2/8 vector for hepatic gene transfer of human apoE3 to inhibit atherosclerotic lesion development in apoE-deficient mice. Atherosclerosis *204*, 121–126.

13. Wu, Z., Sun, J., Zhang, T., Yin, C., Yin, F., Van Dyke, T., Samulski, R.J., and Monahan, P.E. (2008). Optimization of self-complementary AAV vectors for liver-directed expression results in sustained correction of hemophilia B at low vector dose. Mol. Ther. *16*, 280–289.

14. McCarty, D.M., Monahan, P.E., and Samulski, R.J. (2001). Self-complementary recombinant adeno-associated virus (scAAV) vectors promote efficient transduction independently of DNA synthesis. Gene Ther. *8*, 1248–1254.

15. Zhong, S., Sun, S., and Teng, B.B. (2004). The recombinant adeno-associated virus vector (rAAV2)-mediated apolipoprotein B mRNA-specific hammerhead ribozyme: a self-complementary AAV2 vector improves the gene expression. Genet. Vaccines Ther. *2*, 5.

16. Yang, Y.W., and Hsieh, Y.C. (2001). Protamine sulfate enhances the transduction efficiency of recombinant adeno-associated virus-mediated gene delivery. Pharm. Res. *18*, 922–927.

17. Mitchell, A.M., Li, C., and Samulski, R.J. (2013). Arsenic trioxide stabilizes accumulations of adeno-associated virus virions at the perinuclear region, increasing transduction in vitro and in vivo. J. Virol. *87*, 4571–4583.

18. Johnson, J.S., and Samulski, R.J. (2009). Enhancement of adeno-associated virus infection by mobilizing capsids into and out of the nucleolus. J. Virol. *83*, 2632–2644.

19. Russell, D.W., Alexander, I.E., and Miller, A.D. (1995). DNA synthesis and topoisomerase inhibitors increase transduction by adeno-associated virus vectors. Proc. Natl. Acad. Sci. USA *92*, 5719–5723.

20. Chapin, J., Rottensteiner, H., Scheiflinger, F., and Monahan, P.E. (2017). SHP648: an analysis of bleeding rates and factor IX consumption in the phase I/II BAX 335 gene therapy trial in subjects with Hemophilia B. Res. Pract. Thromb. Haemost. *2017* (*Suppl 1*), 144–145.

21. U.S. pharmacopeia (2011). Gene Therapy Products, Potency. Second Supplement to USP 34-NF 29 (U.S. pharmacopeia), https://www.uspnf.com/official-text/proposal-statuscommentary/usp-34-nf-29.

22. European Medicines Agency (2019). Guideline on quality, non-clinical and clinical requirements for investigational advanced therapy medicinal products in clinical trials. January 31, 2019. Committee for Advanced Therapies (CAT), EMA/CAT/852602/2018, https://www.ema.europa.eu/en/documents/scientific-guideline/draft-guideline-quality-non-clinical-clinical-requirements-investigational-advanced-therapy_en.pdf.

23. Gavin, D.K. (2015). Advanced topics: successful development of quality cell and gene therapy products. Food and Drug Administration, Center for Biologics Evaluation and Research Web Seminar Series, https://www.fda.gov/media/80404/download.

24. Liew, L.P., Lim, Z.Y., Cohen, M., Kong, Z., Marjavaara, L., Chabes, A., and Bell, S.D. (2016). Hydroxyurea-Mediated Cytotoxicity Without Inhibition of Ribonucleotide Reductase. Cell Rep. *17*, 1657–1670.

25. Ju, X.D., Lou, S.Q., Wang, W.G., Peng, J.Q., and Tian, H. (2004). Effect of hydroxyurea and etoposide on transduction of human bone marrow mesenchymal stem and progenitor cell by adeno-associated virus vectors. Acta Pharmacol. Sin. *25*, 196–202.

26. Nicolson, S.C., Li, C., Hirsch, M.L., Setola, V., and Samulski, R.J. (2016). Identification and Validation of Small Molecules That Enhance Recombinant Adeno-associated Virus Transduction following High-Throughput Screens. J. Virol. *90*, 7019–7031.

27. Stahnke, S. (2009). Function of the viral phospholipase A2 during adeno-associated virus 2 infection (Die Funktion der viralen Phospholipase A2 während der Infektion des Adeno-assoziierten Virus 2) (PhD thesis University of Cologne).

28. Mitchell, A.M., and Samulski, R.J. (2013). Mechanistic insights into the enhancement of adeno-associated virus transduction by proteasome inhibitors. J. Virol. *87*, 13035–13041.

29. Monahan, P.E., Sun, J., Gui, T., Hu, G., Hannah, W.B., Wichlan, D.G., Wu, Z., Grieger, J.C., Li, C., Suwanmanee, T., et al. (2015). Employing a gain-of-function factor IX variant R338L to advance the efficacy and safety of hemophilia B human gene therapy: preclinical evaluation supporting an ongoing adeno-associated virus clinical trial. Hum. Gene Ther. *26*, 69–81.

30. Yan, C., Costa, R.H., Darnell, J.E., Jr., Chen, J.D., and Van Dyke, T.A. (1990). Distinct positive and negative elements control the limited hepatocyte and choroid plexus expression of transthyretin in transgenic mice. EMBO J. *9*, 869–878.

31. Haut, D.D., and Pintel, D.J. (1998). Intron definition is required for excision of the minute virus of mice small intron and definition of the upstream exon. J. Virol. *72*, 1834–1843.

32. Nathwani, A.C., Gray, J.T., Ng, C.Y., Zhou, J., Spence, Y., Waddington, S.N., Tuddenham, E.G., Kemball-Cook, G., McIntosh, J., Boon-Spijker, M., et al. (2006). Self-complementary adeno-associated virus vectors containing a novel liver-specific human factor IX expression cassette enable highly efficient transduction of murine and nonhuman primate liver. Blood *107*, 2653–2661.

33. Horling, F., Falkner, F.G., Chuah, M., Lengler, J., Mayrhofer, J., Klugmann, M., Coulibaly, S., Hoellriegel, W., Monahan, P.E., Vandendriessche, T., et al. (2017). SHP648: A high performing next generation FIX gene therapy vector based on AAV8. Res Pract Thromb Haemost *2017* (*Suppl 1*), 173–174.

34. Grieger, J.C., Soltys, S.M., and Samulski, R.J. (2016). Production of Recombinant Adeno-associated Virus Vectors Using Suspension HEK293 Cells and Continuous Harvest of Vector From the Culture Media for GMP FIX and FLT1 Clinical Vector. Mol. Ther. *24*, 287–297.

35. Hernandez-Bort, J., Fiedler, C., Buengener, C., Mayrhofer, J., Lengler, J., and Schlager, R. (2018). Biological activity of additional AAV subpopulation in AAV gene therapy product. Human Gene Therapy *29*, A44.

36. Aurnhammer, C., Haase, M., Muether, N., Hausl, M., Rauschhuber, C., Huber, I., Nitschko, H., Busch, U., Sing, A., Ehrhardt, A., and Baiker, A. (2012). Universal real-time PCR for the detection and quantification of adeno-associated virus serotype 2-derived inverted terminal repeat sequences. Hum. Gene Ther. Methods *23*, 18–28.

37. Fagone, P., Wright, J.F., Nathwani, A.C., Nienhuis, A.W., Davidoff, A.M., and Gray, J.T. (2012). Systemic errors in quantitative polymerase chain reaction titration of self-complementary adeno-associated viral vectors and improved alternative methods. Hum. Gene Ther. Methods *23*, 1–7.

38. Lin, H.F., Maeda, N., Smithies, O., Straight, D.L., and Stafford, D.W. (1997). A coagulation factor IX-deficient mouse model for human hemophilia B. Blood *90*, 3962–3966.

39. Dietrich, B., Schiviz, A., Hoellriegl, W., Horling, F., Benamara, K., Rottensteiner, H., Turecek, P.L., Schwarz, H.P., Scheiflinger, F., and Muchitsch, E.M. (2013). Preclinical safety and efficacy of a new recombinant FIX drug product for treatment of hemophilia B. Int. J. Hematol. *98*, 525–532.

OMTM, Volume *17*

Supplemental Information

Development of an *In Vitro* Biopotency Assay for an AAV8 Hemophilia B Gene Therapy Vector Suitable for Clinical Product Release

Johannes Lengler, Sogue Coulibaly, Bernadette Gruber, Reinhard Ilk, Josef Mayrhofer, Friedrich Scheiflinger, Werner Hoellriegl, Falko G. Falkner, and Hanspeter Rottensteiner

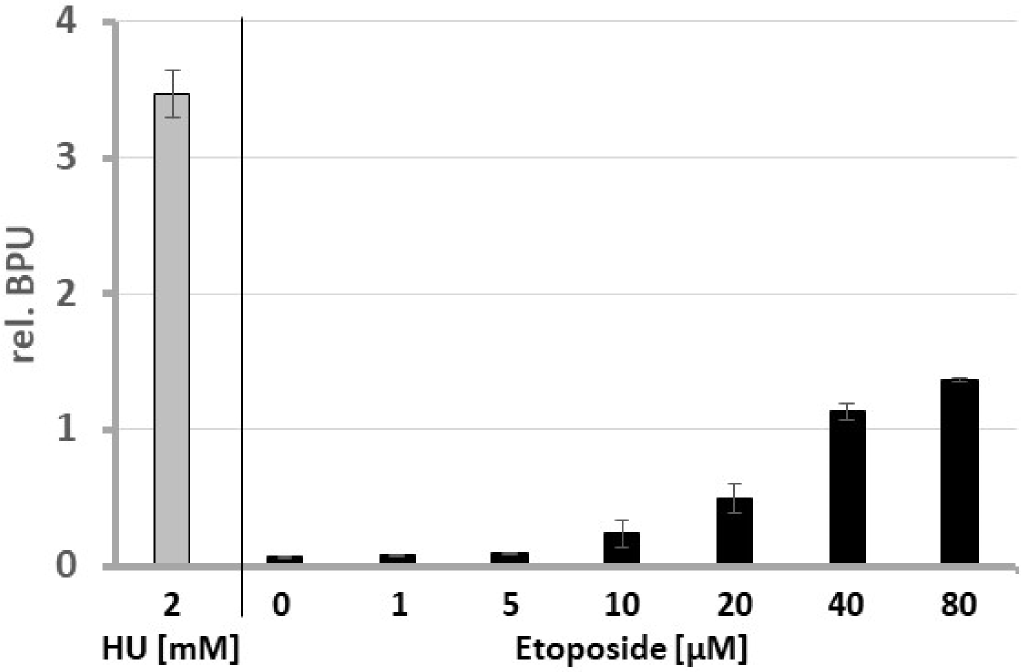

Supplementary Figure 1: Effect of etoposide on the *in vitro* biopotency assay for BAX 335. HepG2 cells were infected with the AAV8-FIX vector (MOI: $3x10^3$) in the presence of increasing concentrations of etoposide or 2 mM HU. After 4 days, FIX activity was measured in the supernatant. The observed increase in FIX activity with higher etoposide concentrations showed saturation at 80 µM and was less pronounced than that obtained with 2 mM HU.

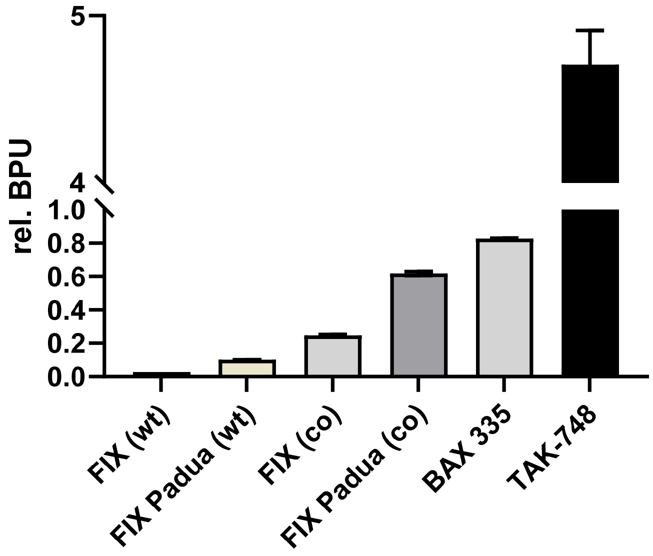

Supplementary Figure 2. Testing of other AAV8-FIX vectors with the *in vitro* biopotency assay for BAX 335. HepG2 cells were infected with the indicated AAV8-FIX vectors at a MOI dosage of $3x10^3$. After 4 days, FIX activity was measured in the supernatant. The expression cassettes of the 'wt' pair of vectors resemble that of the self-complementary vector BAX 335, but contain the normal human *F9* coding sequence, with or without the FIX Padua mutation. The expression cassettes of the 'co' vector pair are identical to those of the 'wt' pair, but contain a codon optimized and CpG-depleted *F9* coding sequence. The second generation AAV8-FIX vector TAK-748 has a single-stranded genome, a codon optimized and CpG-depleted *F9*-Padua coding sequence, and a novel liver-specific promoter. co, codon optimized; MOI, multiplicity of infection; Rel. BPU, relative biopotency unit; wt, wild-type.

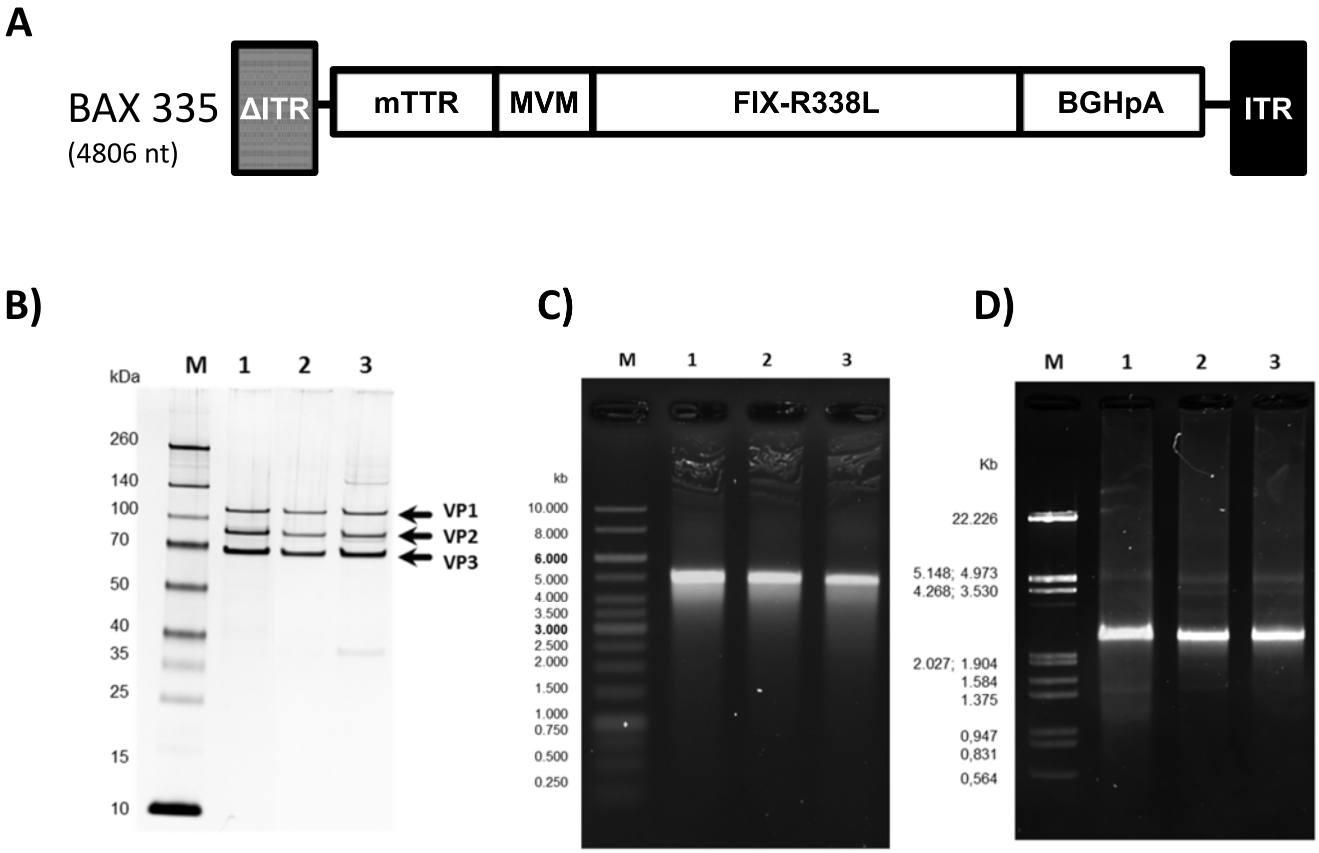

Supplementary Figure 3: Structural analysis of three lots of BAX 335. (A) Schematic drawing of the BAX 335 (scAAV8-FIX-R338L) self-complementary vector genome. **(B)** SDS-PAGE followed by silver staining. Analysis of three BAX 335 lots revealed the expected pattern of capsid proteins VP1, VP2 and VP3. **(C)** Alkaline agarose gel electrophoresis. Shown is the melted vector genome of the same three lots, with a single-stranded size of about 4.8 kb. **(D)** Native agarose gel electrophoresis. Shown are the vector genomes of the same three lots, running as a double stranded entity at a size of about 2.4 kb. The vector preparations show high purity and vector genome integrity. Lane M denotes respective size markers.

Supplementary Table 1: Influence of sample matrix components on *in vitro* biopotency assay specificity

Formulation buffer [%]	Mean BPU (n=2) Run 1	% Recovery	Mean BPU (n=2) Run 2	% Recovery	Mean BPU (n=2) Run 3	% Recovery	Mean BPU (n=6) All runs	% Recovery
1.25	0.84	99.1	0.89	85.6	0.82	92.7	0.85	92
1.00	0.84	99.1	0.89	86.2	0.78	88.2	0.84	90.8
0.75	0.86	101.1	0.96	93.1	0.88	99.9	0.9	97.7
0.50	0.93	109.6	0.97	93.8	0.91	102.7	0.94	101.5
0.25	0.99	116.3	0.92	88.5	0.83	93.9	0.91	98.8
0.00	0.85	100.0	1.03	100	0.88	100.0	0.92	100.0

Supplementary Table 2: Linearity of *in vitro* biopotency assay reference curve

Run	R^2
1	0.9933
2	0.9941
3	0.9964
4	0.9980
5	0.9960
6	0.9978
7	0.9900
8	0.9992
9	0.9945
10	0.9939
11	0.9981
12	0.9902
13	0.9979
14	0.9982
15	1.000
mean	0.9958
SD	0.0031

Supplementary Table 3a: Influence of different WCBs and cell passages on *in vitro* biopotency*

		BPU of control 1		Acceptance criteria [BPU]	
Day of cultivation	Cell passage number	WCB 1 (CS308)	WCB 2 (GR202)	Lower limit: 0.3063	Upper limit: 0.8614
11	13	0.51	0.56	pass	
18	15	0.40	0.59	pass	
25	17	n.a.	0.61	pass	
46	23	0.74	n.a.	pass	
53	25	0.56	0.55	pass	
60	27	0.61	n.a.	pass	

*Infection with small-scale AAV8-FIX vectors

Supplementary Table 3b: Influence of different WCBs and cell passages on *in vitro* biopotency*

		BPU of control 2		Acceptance criteria [BPU]	
Day of cultivation	Cell passage number	WCB 1 (CS308)	WCB 2 (GR202)	Lower limit: 0.3434	Upper limit: 0.7316
11	13	0.50	0.48	pass	
18	15	0.43	0.46	pass	
25	17	n.a.	0.49	pass	
46	23	0.59	n.a.	pass	
53	25	0.46	0.49	pass	
60	27	0.57	n.a.	pass	

*Infection with crude AAV8-FIX conditioned medium

Supplementary Table 3c: Influence of hydroxyurea on *in vitro* biopotency assay

BPU of control 1		Acceptance criterion [BPU]	
HU Lot 1	HU Lot 2	Lower limit: 0.3063	Upper limit: 0.8614
0.60	0.78	pass	
0.61	0.61	pass	
0.62	0.60	pass	
0.65	0.62	pass	

Supplementary Table 3d: Influence of hydroxyurea on *in vitro* biopotency assay

BPU of control 2		Acceptance criterion [BPU]	
HU Lot 1	HU Lot 2	Lower limit: 0.3434	Upper limit: 0.7316
0.47	0.56	pass	
0.46	0.49	pass	
0.47	0.52	pass	
0.50	0.51	pass	
